# Prevalence of a history of violence and domestic violence during pregnancy in Iceland and related risk factors during the COVID-19 pandemic: a cross-sectional study

**DOI:** 10.1186/s12884-025-08049-2

**Published:** 2025-09-08

**Authors:** Hafrún Rafnar Finnbogadóttir, Ástþóra Kristinsdóttir, Rannveig Sigurvinsdóttir, Linda Bára Lýðsdóttir

**Affiliations:** 1 Faculty of Health and Life Sciences, Department of Health and Caring Sciences, Linnaeus University, Kalmar, Sweden; 2 Development Centre for Primary Healthcare in Iceland, Reykjavík, Iceland; 3 Department of Psychology, Reykjavik University, Reykjavík, Iceland

**Keywords:** Anxiety symptoms, COVID-19, Cross-sectional study, Domestic violence, Depressive symptoms, History of violence, Pregnancy

## Abstract

**Background:**

One in three women worldwide will experience physical and/or sexual violence in their lifetime, and pregnancy is a risk factor for domestic violence. Recent studies have identified global stressors, such as the COVID-19 pandemic, as being connected to an increased prevalence of domestic violence. The aim of the present study was threefold: Firstly, to investigate the prevalence of DV among pregnant women during the COVID-19 pandemic in Iceland. Secondly, to examine a history of violence experienced by pregnant women in Iceland. Thirdly, to explore the association of symptoms of anxiety and depression, demographic factors and exposure to violence among pregnant women in Iceland.

**Methods:**

A cross-sectional design was used. A total of 660 pregnant women receiving prenatal care were recruited and completed a survey between April and June 2021. Descriptive statistics were assessed using univariate analysis, Fisher’s exact test, independent t-tests and linear regression analysis as appropriate.

**Results:**

In all, 45.8% (*n =* 302) women were survivors of any type of emotional, physical and/or sexual violence, either in adulthood (*n =* 212, 31.4%) or childhood (*n =* 207, 21.1%). Twenty-three (3.5%) reported abuse during the last 12 months, of which 20 reported domestic violence. Twelve (1.8%) reported abuse during current pregnancy. Exposure to violence in adulthood was associated with unemployment and exposure to violence in childhood was associated with unemployment and lower educational level. Abuse during pregnancy was associated with being single. Regardless of when the experience of violence occurred, depression and anxiety symptoms were more prevalent among women who had experienced violence compared to those who had no such history.

**Conclusions:**

Pregnant Icelandic women commonly reported history of violence, which may be related to their poorer mental health. Iceland benefits from well-organised and comprehensive perinatal services, which need to consider the mental health of women to provide trauma-informed care. Present study did not identify an elevated risk of domestic violence during the COVID-19 pandemic among pregnant women. Still, pregnant women are exposed to domestic violence during pregnancy and there is a need for more resources for research about violence to find effective preventive measures.

## Background

Violence against women (VAW) is a global violation of human rights, and roughly one in three women worldwide experience physical and/or sexual violence in their lifetimes [1]. A history of violence is defined as experiencing emotional, physical, or sexual violence as a child (< 18 years) or as an adult (≥ 18 years), regardless of the severity of the violence or the relationship to the perpetrator [2]. Pregnant women also experience violence, including domestic violence (DV), which covers all forms of abuse within a household or by a person very well known in the family or a significant other, not only limited to intimate relationships [3]. Intimate partner violence (IPV) during pregnancy is, in 90% of cases, perpetrated by the unborn baby’s biological father [4], whether they are married, cohabiting, dating, or former partners, and refers to the same action described for DV [3].

A recent systematic review and meta-analysis of 118 studies from 52 countries showed that 25% of pregnant women worldwide experience IPV [5]. A significant difference in prevalence rates was found between continents, with Africa having the highest prevalence rate and Europe having the lowest (36.1% and 5.1%, respectively). The Nordic countries have relatively low rates of IPV experienced by pregnant women, with the highest prevalence rate being Denmark’s 8.5% [6]. The prevalence rate is 5% in Norway [7], and 2.5% in Sweden [8]. A similar prevalence rate has been found for Icelandic pregnant women; over the last 12 months, 3.3% of them have experienced abuse [9]. Pregnant women in the Nordic countries of Denmark, Iceland, Norway, and Sweden report a history of violence of 33.6%, 35.5%, 37.1% [9], and 39.5%, respectively [2].

Demographic variables contribute to DV risk during pregnancy [10]. These variables include being single, having a low level of education and having low socioeconomic status. Global stressors, such as the COVID-19 pandemic, may increase the prevalence of DV. For example, a scoping review by Kotlar et al. [11] found a spike in DV during the first nine months of the pandemic. Furthermore, a globally focused systematic review and meta-analysis suggested that DV increased or intensified in response to stay-at-home and lockdown orders, which forced families to coexist in the same spaces [11, 12]. In addition, a separate systematic review and meta-analysis found that the prevalence of violence against pregnant women during the COVID-19 epidemic was relatively high, with the pooled prevalence rates of psychological, physical and sexual violence against pregnant women during this time being 24%, 14%, and 6%, respectively [13].

Exposure to violence threatens maternal and foetal health [14] and can result in poorer physical and mental health issues [14–17]. For example, it may heighten the risk of pregnant women developing a fear of childbirth [18–20] or having an emergency or planned Caesarean section [21–23]. Experiencing DV is associated with depression during the perinatal period [24]. Moreover, mothers reporting emotional, physical or sexual abuse have an increased risk of postpartum depression [25]. COVID-19 can have detrimental effects on the health of pregnant women, both directly through the infection itself and indirectly due to the strains COVID-19 has caused on the healthcare systems and economies of various countries [11]. The social and economic impacts of COVID-19 result in adverse effects on maternal health, as they can lead to mental health problems, such as depression and anxiety. During the pandemic, pregnant women experienced dramatically increased worries not only for their own health but also for the health of their unborn children and their partners [26]. Anxiety has been linked to adverse pregnancy outcomes and might have long-term effects on health [10, 27].

Antenatal care (ANC) is an important component of healthy pregnancies and usually involves regular visits to midwives in Iceland. International studies show that identifying DV during ANC is extremely important for violence-exposed pregnant women to offer support and intervene when necessary [28–30]. Previous researchers have identified the COVID-19 pandemic as a period of heightened risk of DV [11, 13], but to our knowledge, no information is available about the prevalence of DV among pregnant women in Iceland during the COVID-19 pandemic.

For a decade and a half, Iceland has led the Gender Equality Index by the World Economic Forum [31], yet rates of VAW in the country remain notably high. This paradox, often called the “Nordic paradox,” reflects a global trend where nations with high gender equality indices still tackle significant levels of VAW [32]. Understanding the predictors of VAW is therefore imperative not only for addressing local challenges but also for contributing to broader global efforts to combat VAW. VAW is a universal problem and responsibility of every society in different countries. What may differ are sociocultural factors, health care systems, economy, and laws [33]. While the body of knowledge has been growing since the early 20 s in Nordic countries, research on pregnant women remains notably scarce in some, where studies are either non-existent (Finland) or becoming outdated (Iceland), and there is a need for larger cohorts and longitudinal approaches [33]. Therefore, it is urgent and essential to advance our understanding of the occurrence of VAW, especially among pregnant women, also in a country such as Iceland with a small population. This research can provide insights into effective policy interventions, community-based support systems, and healthcare strategies adapted to this population’s specific needs.

Building on prior literature, this study aims to achieve three objectives. Firstly, to investigate the prevalence of DV among pregnant women during the COVID-19 pandemic in Iceland. Secondly, to examine a history of violence experienced by pregnant women in Iceland. Thirdly, to explore the association of symptoms of anxiety and depression, demographic factors and exposure to violence among pregnant women in Iceland. Our hypothesis posits that pregnant women with a history of violence will report higher levels of depression and anxiety symptoms compared to those who have not experienced such violence.

## Methods

This is a multi-center, nationwide study with a cross-sectional design and prospectively collected data.

### Participants and procedure

A total of 660 pregnant women participated in the study; they met the inclusion criteria of being pregnant, at least 18 years of age, receiving ANC within primary healthcare settings, and being able to read and understand Icelandic. The participants were pregnant women who attended prenatal examinations for three months between April and June 2021 at 28 different ANCs in Iceland. Sixteen of these clinics were in the greater Reykjavík area, while the remaining nine were in various regions across Iceland, including the South, West, Westfjords, North, and Eastfjords. Recruitment was carried out by midwives who worked at these ANCs during the three months. In primary health care settings, ANC is offered free of charge to pregnant women, and almost all use these services. Women requiring specialized ANC for complicated pregnancies are referred to the Center for High-Risk Pregnancy at the National University Hospital of Iceland. These women were not included in the present study. During the recruitment period, a total of 1384 pregnant women registered to receive ANC. Specifically, 869 resided in the urban area of Reykjavík, and 515 resided in rural areas. We estimate that 47.7% of women who received ANC during the study period completed the questionnaire, but because we did not carry out recruitment ourselves, it was not possible to determine how many women refused to participate or how many women were simply not informed about the study.

During recruitment, women receiving ANC were given information about the study and asked to provide informed consent before answering any questions. They were assured of their data’s confidentiality and were informed that they were not obligated to disclose to their midwife whether they were involved with an abusive partner. If a woman agreed to participate in the study, she was directed to a private room to complete the questionnaires after completing her examination. Due to COVID-19 restrictions, spouses were generally not allowed to accompany pregnant women to their prenatal examinations. However, in the few cases where a spouse was present during the examination, the spouse was asked to wait outside, or the woman was asked to attend by herself next time. Additionally, if participants became distressed about completing the questionnaire, they were provided with access to a psychologist within the healthcare system.

### Instruments

In addition to the standard measures outlined below, participants completed a questionnaire about their demographic characteristics. They were asked about their age, parity (primiparous or multiparous), educational level (10 years or less, 11–14 years, 15 years or more and university), cohabiting status (single, married or in a relationship) and employment status (employed/student, homemaker or on parental leave, on sick leave, unemployed or on disability benefits).

The *NorVold Abuse Questionnaire* (NorAQ) [34] measures emotional, physical and sexual violence in childhood (< 18 years) and in adulthood (≥ 18 years). In addition, a yes or no question about the experience of violence during the past 12 months was included, followed by a question about the survey respondent’s relationship to the perpetrator and the perpetrator’s gender. The NorAQ has good reliability, validity and specificity [34]. The specificity was 98% (95% confidence interval [CI]) for all kinds of abuse except physical abuse, which was 85%. Sensitivity ranged from 75% (emotional) to 96% (physical). All items pertaining to abuse from the NorAQ questionnaire were administered in their original format to maintain the instrument’s reliability, validity and specificity. The Icelandic version of the NorAQ has been used in Iceland [9].

The *Abuse Assessment Screen* (AAS) [35] is one of the most widely used screening tools for IPV among pregnant women and consists of five short questions. One modified question was used in this study, ‘Have you been exposed to abuse during your current pregnancy?’ to investigate emotional, physical and sexual abuse (yes or no; if yes, by whom). This modified question has been used in previous studies on this topic [2, 8, 36].

The *Edinburgh Postnatal Depression Scale* (EPDS) [37] is one of the most widely used screening tools for depression in the perinatal period. This 10-item self-report questionnaire uses a scoring system of 0 to 3 for each item, with higher scores indicating more symptoms of depression. The scale is a reliable and valid instrument for use in both the antenatal and postpartum periods [38–40]. The Icelandic version of the EPDS has reliability and excellent internal consistency (α = 0.84-0.85) [41]. A cutoff value of 11 was considered optimal, with a sensitivity of 0.80 and a specificity of 0.89 [41]. The internal consistency of the scale in this study was α = 0.85.

The *General Anxiety Disorder Scale* (GAD-7) [42] is a seven-item self-report questionnaire designed to assess the presence and severity of anxiety symptoms. Each item is scored on a scale from 0 to 3, with higher scores indicating more severe symptoms of anxiety. The GAD-7 has excellent internal consistency (α = 0.92) [42]. The Icelandic version of the GAD-7 also has good reliability and excellent internal consistency (α = 0.87), [43]. A cutoff value of 10 is considered optimal, with a sensitivity of 86.2% and a specificity of 95.5% [42]. The internal consistency of the scale in this study was α = *0.87.*

### Data analysis

Statistical analysis was carried out using SPSS Statistics version 27 (IBM Corp.). Statistical power calculations indicated that a sample size of 696 participants was necessary for an accurate statistical analysis. Background variables were classified for descriptive purposes. Parity was categorised as primiparous or multiparous. Education level was classified as primary/secondary education (14 years or less) or tertiary education (15 years or more/university). Marital status was divided into two categories: married/in a relationship and single. Employment status was also classified into two categories: unemployed (including those who were not employed, homemakers or on disability benefits) and employed (including those who were employed, students and on parental leave or sick leave).

A univariate analysis was conducted using Pearson’s chi-squared tests to examine the association between demographic factors and having experienced violence in pregnancy in the last 12 months, in adulthood and in childhood. In the case of small samples (expected frequency lower than five in a cell), Fisher’s exact test was conducted. An independent t-test was conducted to determine any differences in symptoms of depression and anxiety between those who had experienced violence and those who had not. Subsequently, two linear regression analyses were performed using the forced-entry method to determine the relationship between violence in childhood and adulthood and symptoms of depression and anxiety. To improve the reliability of the statistical estimates, both analyses were completed using 1000 bootstrapped resamples and 95% bias-corrected and accelerated (BCa) CIs, which were adjusted in the bootstrap distribution for both bias and skewness and are considered more accurate [44]. Statistical significance was confirmed at *p* <.05 (two-tailed).

## Results

In total, 660 pregnant women with a mean age of 29.90 years (standard deviation [*SD*] = 4.82, range 18–45) participated in the study; most were older than 25 years (*n* = 526, 79.7%). The demographic characteristics of the sample are given in Table 1. Most of the women were married or in a relationship, had completed tertiary education, and were either employed or studying. More than half of them were multiparous. The depression symptom mean score for all participants was 6.4 (*SD* = 4.4), with 18.4% (*n* = 118) scoring above the cutoff score of 11. The anxiety symptom mean score was 4.8 (*SD* = 3.7), with 10.9% (*n* = 65) scoring above the cutoff score of 10.

**Table 1:** Demographic information(*N* = 660)

	Number (%) *
Paritet (*n* = 659)	
Multiparae	372 (56.4)
Primiparae	287 (43.6)
Marital status (*n* = 658)	
Single	14 (2.1)
In relationship	485 (73.7)
Married	159 (24.2)
Educational status (*n* = 657)	
≤10 years	22 (3.3)
11–14 years	121(18.4)
≥15 years	123 (18.7)
University degree	391 (59.5)
Employment status (*n* = 658)	
Working	469 (71.3)
Studying	79 (12.0)
Both working and studying	38 (5.8)
Homemaker	6 (0.9)
Unemployed	24 (3.6)
Pregnancy parental leave	18 (2.7)
Sickness leave	6 (0.9)
Social benefits	18 (2.8)

*Missing answers are 1–3

### Prevalence of violence and demographic characteristics

Table 2 shows a detailed breakdown of the prevalence of emotional, physical and sexual violence at any point in the participants’ lives. Almost half of the women who participated in the study reported having experienced violence (45.8%). Sexual violence was the most common type, with high severity for around one-fifth of the participants. Experiences of IPV from a previous partner were more common than experiences of IPV from a current spouse. Furthermore, recent experiences of violence were rare, as only 23 women reported violence in the past year and 12 during their current pregnancy, 8 of which had experienced DV.

**Table 2:** Lifetime prevalence of emotional, physical, and sexual abuse reported by pregnant women* (*N* = 660)

Abuse	Lifetime abuse *n* (%) 302 (45.8)	In childhood *n* (%) 212 (32.1)	In adulthood *n* (%) 207 (31.4)	Last 12 months *n* (%) 23 (3.5)	During pregnancy *n* (%) 12 (1.8)	IPV Current spouse *n* (%) 13 (2.0)	IPV Ex-spouse *n* (%) 134 (20.3)
Emotional abuse	176 (26.7)	123 (18.6)	107 (16.2)	17 (2.6)	9 (1.4)	9 (1.4)	110 (16.7)
Mild	161 (24.5)	111 (16.9)	94 (14.3)				
Moderate	112 (17.1)	55 (8.4)	72 (11.0)				
Severe	72 (10.9)	43 (6.5)	39 (5.9)				
Physical abuse	156 (23.6)	98 (14.8)	87 (13.2)	5 (0.8)	3 (0.5)	6 (0.9)	71 (10.8)
Mild	142 (21.6)	90 (13.7)	77 (11.7)				
Moderate	100 (15.2)	53 (8.0)	59 (9.0)				
Severe	49 (6.1)	17 (2.6)	28 (4.3)				
Sexual abuse	215 (32.6)	139 (21.1)	139 (21.1)	4 (0.6)	1 (0.2)	2 (0.3)	58 (8.8)
Mild: no genital contact	179 (27.2)	104 (15.8)	114 (17.3)				
Mild: emotional sexual humiliation	48 (7.4)	31 (4.8)	20 (3.1)				
Moderate: genital contact	176 (26.9)	108 (16.5)	102 (15.6)				
Severe: penetration	141 (21.5)	70 (10.7)	92 (14.0)				

*Categories are not mutually exclusive, so the same woman may have reported several types of abuse

Table 3 shows the relationships between experiences of violence and demographic factors. The chi-squared test results showed that women who had experienced violence in childhood and adulthood were more likely to be unemployed and that childhood exposure to violence was associated with a lower educational level. Single women were significantly more likely to have been exposed to violence, as indicated by Fisher’s exact test (Table 3).

**Table 3:** The association between demographic factors and experienced violence in pregnancy, in the last 12 months, in adulthood, and in childhood in pregnant women seeking maternity care during COVID-19 (*N* = 660)

	Pregnancy *n* (%)		Last 12 months		Adulthood *n* (%)		Childhood *n* (%)	
	Abuse *n* (%)	No abuse *n* (%)	Abuse *n* (%)	No abuse *n* (%)	Abuse	No abuse	Abuse	No abuse
Age								
18–25	4 (33.3)	121 (18.9)	4 (17.4)	121 (19.3)	33 (16.3)	92 (20.5)	46 (22.1)	79 (17.8)
>25	8 (66.7)	518 (81.1)	19 (82.6)	507 (80.7)	169 (83.7)	357 (79.5)	162 (77.9)	364 (82.2)
Parity								
Primiparae	5 (41.7)	282 (43.6)	8 (34.8)	279 (43.9)	86 (41.7)	201 (44.4)	100 (47.4)	187 (41.7)
Multiparae	7 (58.3)	365 (56.4)	15 (65.2)	359 (56.1)	120 (58.3)	252 (55.6)	111 (52.6)	261 (58.3)
Marital status								
Single	2 (16.7) *	12 (1.9)	2 (9.1)	11 (1.9)	4 (2)	10 (2.2)	7 (3.3)	7 (1.6)
Married/cohabitating	10 (83.3)	626 (98.1)	20 (90.9)	554 (98.1)	198 (98)	438 (97.8)	202 (96.7)	434 (98.4)
Education level								
≤14 years	5 (41.7)	138 (21,4)	7 (30.4)	136 (21.5)	53 (25.7)	90 (20)	68 (32.4) **	75 (16.8)
≥15 years	7 (58.3)	507 (78.6)	16 (69.6)	498 (78.5)	153 (74.3)	361 (80)	142 (67.6)	372 (83.2)
Employment status								
Unemployed	2 (16.7)	40 (6.8)	4 (18.2)	38 (6.5)	19 (10.3)	23 (5.5) ***	22 (12) ****	20 (4.8)
Employed	10 (83.3)	551 (93.2)	18 (81.8)	543 (93.5)	165 (89.7)	396 (94.5)	162 (88)	399 (95.2)

Fischer exacta test used in case of small figures < 5 * *p* =.025, ** χ2 (1, *N* = 657) = 20.43, *p* <.001, *** χ2 (1, *N* = 603) = 4.62, *p* =.032, **** χ2 (1, *N* = 603) = 10.18, *p* =.001

### Violence and mental health

Table 4 shows the symptoms of depression and anxiety in relation to exposure to violence. Independent samples t-tests showed that regardless of when the experience of violence occurred, depression symptoms were more prevalent among women who had experienced violence. This difference was particularly pronounced when episodes of violence had occurred more recently. A similar pattern emerged for anxiety symptoms, which were significantly more common among women who had experienced violence compared to those who had no such history.

**Table 4:** Symptoms of depression and anxiety by exposure to violence (*N* = 660)

	Depression symptoms						Anxiety symptoms					
	Yes		No		t	d	Yes		No		t	d
	M	SD	M	SD			M	SD	M	SD		
Violence during pregnancy	11.04	6.62	6.26	4.27	3.7**	1.1	9.04	5.64	4.65	3.55	3.70**	1.1
Violence in the last 12 months	11.5	6.04	6.33	4.37	2.95*	1.17	8.58	5.18	4.74	3.67	2.56*	1.17
Violence in adulthood	7.60	4.78	5.90	4.22	4.04**	0.34	6.03	4.22	4.23	3.32	3.93**	0.38
Violence in childhood	7.72	4.88	5.81	4.10	4.90**	0.44	6.03	4.22	4.23	3.32	5.45**	0.5

* *p* <.05, ** *p* <.001

X̄ mean; *SD* standard deviation

Table 5 shows Pearson’s bivariate correlations between depression and anxiety symptoms, demographic characteristics and exposure to violence. Both violence in childhood and adulthood were significantly related to the presence of more depression and anxiety symptoms. Age had a negative relationship with depression and anxiety symptoms. Linear regression analysis (Table 6) showed that age, education level and violence in both childhood and adulthood predicted the occurrence of more depression symptoms (R^2^ = 0.080) and anxiety symptoms (R^2^ = 0.097). Both violence in childhood and adulthood contributed unique variance to the model when demographic factors were controlled for. For both mental health outcomes, each step added significant variance to the model.

**Table 5:** Pearson’s correlations between study variables (*N* = 660)

Variables	*N*	1.	2.	3.	4.	5.	6.	7.	8.	9.
1. Depression	658	1	0.78**	−11**	0.00	0.08	0.17**	0.11**	0.20**	0.16**
2. Anxiety	651		1	0.17**	0.00	0.01	0.18**	0.08*	0.23**	0.16**
3. Age	649			1	0.35**	0.06	0.22**	0.08*	0.05	− 0.05
4. Parity	657				1	0.02	− 0.01	0.01	0.05	− 0.03
5. Marital status	656					1	0.08	0.16**	0.06	− 0.01
6. Educational status	655						1	0.23**	0.18**	0.07
7. Employment status	656							1	0.13**	0.11**
8. Childhood violence	658								1	0.35**
9. Adulthood violence	658									1

R^2^ *p <.05. **p <.01.

**Table 6:** Linear regression predicting depression and anxiety symptoms (*N* = 660)

	Depression symptoms						Anxiety symptoms					
	*B*	*SE*	*β*	*95% CI*		*p*	*B*	*SE*	*β*	*95% CI*		*p*
				*LL*	*UL*					*LL*	*UL*	
Step 1												
Constant	8.09	1.14		5.84	10.33	< 0.001	7.18	0.96		5.31	9.06	< 0.001
Age	− 0.07	0.04	− 0.08	− 0.14	0.00	ns	− 0.09	0.03	− 0.12	− 0.15	− 0.03	0.003
Educational level	1.40	0.45	0.13	0.52	2.27	0.002	1.21	0.37	0.13	0.48	1.94	0.001
Employment status	1.61	0.70	0.09	0.24	2.98	0.021	0.92	0.58	0.06	− 0.21	2.06	Ns
Step 2												
Constant	7.70	1.13		5.48	9.92	< 0.001	6.81	0.94		4.96	8.65	< 0.001
Age	− 0.07	0.04	− 0.08	− 0.14	− 0.00	0.045	− 0.09	0.03	− 0.12	− 0.15	− 0.03	0.002
Educational level	1.10	0.45	0.10	0.23	1.98	0.014	0.93	0.37	0.10	0.21	1.66	0.012
Employment status	1.28	0.69	0.07	− 0.08	2.64	Ns	0.62	0.57	0.04	− 0.50	1.74	Ns
Abuse in childhood	1.64	0.37	0.17	0.91	2.37	< 0.001	1.59	0.31	0.20	0.98	2.19	< 0.001
Step 3												
Constant	7.86	1.13		5.65	10.08	< 0.001	6.94	0.94		5.10	8.78	< 0.001
Age	− 0.09	0.04	− 0.09	− 0.16	− 0.01	0.021	− 0.10	0.03	− 0.13	− 0.16	− 0.04	< 0.001
Educational level	1.08	0.44	0.10	0.21	1.95	015	0.92	0.37	0.10	0.19	1.64	0.013
Employment status	1.17	0.69	0.07	− 0.19	2.53	Ns	0.53	0.57	0.04	− 0.58	1.64	Ns
Violence in childhood	1.29	0.39	0.14	0.51	2.06	0.001	1.28	0.33	0.16	0.64	1.92	< 0.001
Violence in adulthood	1.02	0.39	0.11	0.24	1.79	0.010	0.88	0.33	0.11	24	1.53	0.007

Confidence intervals (CI) and standard errors are based on 1000 bootstrap samples. Depression: R^2^ = 0.042 for step 1; ΔR^2^ = 0.028 for Step 2 (*p* <.001); ΔR^2^ = 0.010 for Step 3 (*p* =.010); anxiety: R^2^ = 0.049 for step 1; ΔR^2^ = 0.038 for Step 2 (*p* <.001); ΔR^2^ = 0.010 for Step 3 (*p* =.007)

## Discussion

The study revealed a low reported prevalence of violence during pregnancy, with only 1.8% of women reporting exposure to violence during their current pregnancy and 3.5% exposed within the last 12 months. This contradicts other studies that identified spikes in DV rates during the pandemic [11–13]. However, there was a significant difference in how different countries handled the COVID-19 pandemic, which may have influenced these prevalence rates, e.g. lockdowns and restrictions were milder in Iceland than in many other countries. However, the response rate of the participants could have been better, as this may have impacted the prevalence.

In the present study, a history of violence was commonly reported by pregnant Icelandic women. Nearly one in two pregnant women reported experiencing some form of violence during their childhood or as adults. This figure reflects an increase of more than 10% compared to earlier findings in a similar-sized cohort from Iceland using the same instrument for revealing experiences of violence [9]. The prevalence of a history of violence in the present study is more in line with rates from a Swedish study where 4 out of 10 women among a cohort of almost 2000 pregnant women had experienced violence [2]. It is unclear why the history of violence is more prevalent among pregnant Icelandic women today than about one decade ago. Possible explanations include more open societal discourse on violence, allowing women greater opportunity to name their experiences as violence. It may also be more permissible and less shameful to admit to victimisation today than before, perhaps in part due to the #MeToo movement [45].

About seven out of ten women who had experienced violence reported having experienced sexual abuse, and more than half of those women (65.6%) had experienced severe sexual abuse (with penetration). This figure is substantially higher than the 16.3% estimate identified in a study conducted between 2008 and 2010 among pregnant Icelandic women where the same instrument was used [9].

The literature has shown that experiences of violence confer unique risks during the prenatal period. For example, a history of sexual violence is associated with greater fear of childbirth [18–20]. A multicentre study of six European countries showed that women who report severe fear of childbirth are significantly more likely to give birth by elective Caesarean section, [46]. A history of emotional abuse also significantly increases the risk of having a Caesarean section [23]. International studies support an increased risk for both planned (on request) and emergency Caesarean section [47, 48]. Therefore, midwives and other healthcare personnel working with pregnant women having a history of violence should be aware that this population may be at higher risk of both fear of childbirth and having a Caesarean section. Indeed, all Nordic countries systematically address violence against pregnant women by asking pregnant women when visiting ANC about the history of violence they may carry on and thereby inviting the pregnant women to dialogue about it [33]. This aligns with WHO clinical and policy guidelines about how to respond to IPV [30].

The present study’s results showed that women with a history of violence presented more symptoms of depression and anxiety than women without a history of violence. This difference was particularly pronounced when the violence was more recent. This is in line with previous studies [17, 49]. The demographic variables of younger age, lower education level and abuse in both childhood and adulthood predicted a higher likelihood of depression. A recent Scoping review with 61 papers from the Nordic countries supports worse psychological health if a childbearing woman has a history of violence [33]. Both anxiety and depression are well-known effects of DV during the perinatal period [17, 24, 33, 49]. Anxiety may lead to adverse pregnancy outcomes and long-term effects [27]. Mothers who experience emotional, physical and sexual abuse are also at an increased risk for postpartum depression [25].

The results showed a significant association between exposure to violence during pregnancy and being single. This result has been confirmed by several studies [8, 10]. Being young and unemployed and having a low educational level were more common among women who had been exposed to violence, but this association was not significant. Still, these risk factors are well-known in the literature [10]. However, because of the small sample size in the present study, a careful conclusion should be made about such an association.

The present study’s findings suggest that although the COVID-19 pandemic did not confer additional risk of violence to pregnant Icelandic women, they have trauma histories that may impact their pregnancy and well-being more generally. Therefore, health professionals and governmental agencies need to put a special focus on providing trauma-informed care for pregnant women and determining ways to best meet their needs during this critical stage in their lives. To ensure that pregnant women who have been exposed to violence are comfortable disclosing both current victimisation and their histories of trauma during ANC, it is crucial to offer therapy or other assistance to avoid negative outcomes, both for the mother and the baby. Two studies from the Nordic countries have shown that pregnant women with a history of violence commonly suffer and may experience unprocessed negative emotions related to their past trauma [9, 50]. Indeed, it is the responsibility of midwives and other healthcare professionals to identify women with a trauma history and offer them appropriate resources.

### Strengths and limitations

This study has several strengths stemming from using validated instruments to collect data [34, 35, 37, 42]. Additionally, the sample comprised unselected pregnant women visiting ANC at primary health care, and the study featured a multicentre national design. However, one significant limitation of our study is excluding women receiving specialized care for complicated pregnancies. These women were referred to the Center for High-Risk Pregnancy at the National University Hospital of Iceland, which did not participate in our study. Consequently, cases of specialized ANC for complicated pregnancies were not targeted for recruitment, which is seen as a weakness in the study design. In fact, women who have been exposed to violence are more likely to have complicated pregnancies, with hospitalisations [51], premature birth and small gestational age [14] being possible issues. Therefore, the exclusion of this group could have led to an underestimation of the prevalence of violence perpetrated against pregnant Icelandic women. Future research should aim to include women from specialized ANC settings to provide a more comprehensive understanding of violence during pregnancy. Furthermore, the low response rate and uncertainty regarding how many women were asked to participate in the study are limitations. Additionally, the data collected were cross-sectional, making it difficult to infer causality in the relationships identified. Recruitment for the study was negatively impacted by strained working conditions at the ANC centres due to the COVID-19 pandemic, as well as summer vacations during the third month of the recruitment to the study. The study exclusively targeted Icelandic-speaking women, since translating the questionnaire into other languages was impossible. This is a significant limitation, as the proportion of immigrants living in Iceland in 2022 was 16% of the population [52]. Nevertheless, it is important to identify exposure to violence early in pregnancy and to offer individually adapted support to ensure the best possible outcomes for the mother and the child. The literature has supported this issue’s importance [28–30].

## Conclusions

Contrary to prior literature, the present study did not identify an elevated risk of DV during the COVID-19 pandemic among pregnant Icelandic women. However, the study revealed that almost every other pregnant woman surveyed had experienced violence and were carrying on history of violence, an alarming fact given the well-known consequences of violence on the health outcomes of both mothers and babies. Therefore, it is of utmost necessity to provide healthcare personnel with resources not only for identifying these women as a first step prior to intervention but also for educating and supporting midwives and other healthcare professionals to handle this delicate topic. Furthermore, efforts should be made in society to prevent VAW. This includes allocating more resources towards research to identify effective preventive measures.

## Data Availability

No datasets were generated or analysed during the current study.

## Abbreviations

AAS: Abuse assessment screen
ANC: Antenatal care
BCa: Bias-corrected and accelerated
CI: Confidence interval
DV: Domestic violence
EPDS: Edinburg Postpartum Depression Scale
GAD: The General Anxiety Disorder Scale
IPV: Intimate partner violence
NorAQ: The NorVold Abuse Questionnaire
SD: Standard deviation
VAW: Violence against women
WHO: World Health Organization

## References

[1] WHO. Violence against women prevalence estimates, 2018: global, regional and national prevalence estimates for intimate partner violence against women and global and regional prevalence estimates for non-partner sexual violence against women. Executive summary. In. Geneva; 2021.

[2] Finnbogadóttir H, Dykes A-K, Wann-Hansson C. Prevalence of domestic violence during pregnancy and related risk factors: a cross-sectional study in Southern Sweden. BMC Womens Health. 2014;14(1):63–63. 24885532 10.1186/1472-6874-14-63 PMC4030020

[3] Krug EG. World report on violence and health. Geneva: World Health Organization; 2002.

[4] Garcia-Moreno C, Jansen HA, Ellsberg M, Heise L, Watts CH. Prevalence of intimate partner violence: findings from the WHO multi-country study on women’s health and domestic violence. Lancet. 2006;368(9543):1260–9. 17027732 10.1016/S0140-6736(06)69523-8

[5] Roman-Galvez RM, Martin-Pelaez S, Fernandez-Felix BM, Zamora J, Khan KS, Bueno-Cavanillas A. Worldwide prevalence of intimate partner violence in pregnancy. A systematic review and meta-analysis. Front Public Health. 2021;9: 738459. 34527656 10.3389/fpubh.2021.738459 PMC8435609

[6] Andreasen K, Zapata-Calvente AL, Martín-de-Las-Heras S, Bueno-Cavanillas A, Schei B, Dokkedahl S, de León de León S, Fernandez Lopez R, Oviedo-Gutiérrez A, Ankerstjerne LBS, et al. Video consultations and safety app targeting pregnant women exposed to intimate partner violence in Denmark and spain: nested cohort intervention study (STOP study). JMIR Form Res. 2023;7:e38563. 36939835 10.2196/38563 PMC10132014

[7] Sørbø MF, Grimstad H, Bjørngaard JH, Schei B, Lukasse M. Prevalence of sexual, physical and emotional abuse in the Norwegian mother and child cohort study. BMC Public Health. 2013;13(1):186–186. 23452504 10.1186/1471-2458-13-186 PMC3599849

[8] Finnbogadottir H, Dykes AK. Increasing prevalence and incidence of domestic violence during the pregnancy and one and a half year postpartum, as well as risk factors: - a longitudinal cohort study in Southern Sweden . BMC Pregnancy Childbirth. 2016;16:327. 10.1186/s12884-016-1122-6 PMC5081903 27784283

[9] Lukasse M, Schroll A-M, Ryding EL, Campbell J, Karro H, Kristjansdottir H, Laanpere M, Steingrimsdottir T, Tabor A, Temmerman M, et al. Prevalence of emotional, physical and sexual abuse among pregnant women in six European countries. Acta Obstet Gynecol Scand. 2014;93(7):669–77. 24720803 10.1111/aogs.12392

[10] James L, Brody D, Hamilton Z. Risk factors for domestic violence during pregnancy: a meta-analytic review. Violence Vict. 2013;28(3):359–80. 23862304 10.1891/0886-6708.vv-d-12-00034

[11] Kotlar B, Gerson EM, Petrillo S, Langer A, Tiemeier H. The impact of the COVID-19 pandemic on maternal and perinatal health: a scoping review. Reprod Health. 2021;18(1): 10. 33461593 10.1186/s12978-021-01070-6 PMC7812564

[12] Piquero AR, Jennings WG, Jemison E, Kaukinen C, Knaul FM. Domestic violence during the COVID-19 pandemic - evidence from a systematic review and meta-analysis. J Criminal Justice. 2021;74: 101806. 10.1016/j.jcrimjus.2021.101806 PMC9582712 36281275

[13] Huldani H, Kamal Abdelbasset W, Abdalkareem Jasim S, Suksatan W, Turki Jalil A, Thangavelu L, Fakri Mustafa Y, Karami M. Intimate partner violence against pregnant women during the COVID-19 pandemic: a systematic review and meta-analysis. Women Health. 2022;62(6):556–64. 35791678 10.1080/03630242.2022.2096755

[14] Donovan BM, Spracklen CN, Schweizer ML, Ryckman KK, Saftlas AF. Intimate partner violence during pregnancy and the risk for adverse infant outcomes: a systematic review and meta-analysis. BJOG. 2016;123(8):1289–99. 26956568 10.1111/1471-0528.13928

[15] Howard LM, Trevillion K, Agnew-Davies R. Domestic violence and mental health. Int Rev Psychiatry. 2010;22(5):525–34. 21047164 10.3109/09540261.2010.512283

[16] Hill A, Pallitto C, McCleary-Sills J, Garcia-Moreno C. A systematic review and meta-analysis of intimate partner violence during pregnancy and selected birth outcomes. Int J Gynaecol Obstet. 2016;133(3):269–76. 27039053 10.1016/j.ijgo.2015.10.023

[17] Howard LM, Oram S, Galley H, Trevillion K, Feder G. Domestic violence and perinatal mental disorders: a systematic review and meta-analysis. PLoS Med. 2013;10(5):e1001452. 23723741 10.1371/journal.pmed.1001452 PMC3665851

[18] Lukasse M, Schei B, Ryding EL, Bidens Study G. Prevalence and associated factors of fear of childbirth in six European countries. Sex Reprod Healthc. 2014;5(3):99–106. 25200969 10.1016/j.srhc.2014.06.007

[19] Henriksen L, Schei B, Lukasse M. Lifetime sexual violence and childbirth expectations - a Norwegian population based cohort study. Midwifery. 2016;36:14–20. 27106939 10.1016/j.midw.2016.02.018

[20] Lukasse M, Vangen S, Oian P, Schei B. Fear of childbirth, women’s preference for Cesarean section and childhood abuse: a longitudinal study. Acta Obstet Gynecol Scand. 2011;90(1):33–40. 21275913 10.1111/j.1600-0412.2010.01024.x

[21] Schei B, Lukasse M, Ryding EL, Campbell J, Karro H, Kristjansdottir H, Laanpere M, Schroll A-M, Tabor A, Temmerman M, et al. A history of abuse and operative delivery–results from a European multi-country cohort study. PLoS ONE. 2014;9(1):1. 10.1371/journal.pone.0087579 PMC3909197 24498142

[22] Henriksen L, Schei B, Vangen S, Lukasse M. Sexual violence and mode of delivery: a population-based cohort study. BJOG. 2014;121(10):1237–44. 24939396 10.1111/1471-0528.12923

[23] Finnbogadóttir H, Baird K, Thies-Lagergren L. Birth outcomes in a Swedish population of women reporting a history of violence including domestic violence during pregnancy: a longitudinal cohort study. BMC Pregnancy Childbirth. 2020;20(1):1–10. 10.1186/s12884-020-02864-5 PMC7098079 32216780

[24] Wikman A, Axfors C, Iliadis SI, Cox J, Fransson E, Skalkidou A. Characteristics of women with different perinatal depression trajectories. J Neurosci Res. 2020;98(7):1268–82. 30723972 10.1002/jnr.24390

[25] Sorbo MF, Grimstad H, Bjorngaard JH, Lukasse M, Schei B. Adult physical, sexual, and emotional abuse and postpartum depression, a population based, prospective study of 53,065 women in the Norwegian mother and child cohort study. BMC Pregnancy Childbirth. 2014;14:316 . 10.1186/1471-2393-14-316 PMC4177252 25199411

[26] Naurin E, Markstedt E, Stolle D, Enström D, Wallin A, Andreasson I, Attebo B, Eriksson O, Martinsson K, Elden H, et al. Pregnant under the pressure of a pandemic: a large-scale longitudinal survey before and during the COVID-19 outbreak. Eur J Public Health. 2021;31(1):7–13. 33231625 10.1093/eurpub/ckaa223 PMC7717243

[27] Dunkel Schetter C, Tanner L. Anxiety, depression and stress in pregnancy: implications for mothers, children, research, and practice. Curr Opin Psychiatry. 2012;25(2):141–8. 22262028 10.1097/YCO.0b013e3283503680 PMC4447112

[28] O’Doherty L, Hegarty K, Ramsay J, Davidson LL, Feder G, Taft A. Screening women for intimate partner violence in healthcare settings. Cochrane Database Syst Rev. 2015;2015(7):Cd007007. 26200817 10.1002/14651858.CD007007.pub3 PMC6599831

[29] Sprague S, Slobogean GP, Spurr H, McKay P, Scott T, Arseneau E, Memon M, Bhandari M, Swaminathan A. A scoping review of intimate partner violence screening programs for health care professionals. PLoS One. 2016;11(12):e0168502. 27977769 10.1371/journal.pone.0168502 PMC5158065

[30] World Health O. Responding to intimate partner violence and sexual violence against women: WHO clinical and policy guidelines. Geneva: World Health Organization; 2013. 24354041

[31] World Economic Forum. Global gender gap report 2022. World Economic Forum; 2024.

[32] Gracia E, Merlo J. Intimate partner violence against women and the Nordic paradox. Soc Sci Med. 2016;157:27–30. 27058634 10.1016/j.socscimed.2016.03.040

[33] Finnbogadóttir HR, Henriksen L, Hegaard HK, Halldórsdóttir S, Paavilainen E, Lukasse M, Broberg L. The consequences of a history of violence on women’s pregnancy and childbirth in the Nordic countries: a scoping review. Trauma Violence Abuse. 2024. 10.1177/15248380241253044. 38805432 10.1177/15248380241253044 PMC11545221

[34] Swahnberg IM, Wijma B. The norvold abuse questionnaire (NorAQ): validation of new measures of emotional, physical, and sexual abuse, and abuse in the health care system among women. Eur J Public Health. 2003;13(4):361–6. 14703325 10.1093/eurpub/13.4.361

[35] McFarlane J, Parker B, Soeken K, Bullock L. Assessing for abuse during pregnancy. Severity and frequency of injuries and associated entry into prenatal care. JAMA. 1992;267(23):3176–8. 1593739 10.1001/jama.267.23.3176

[36] Finnbogadóttir H, Dykes A-K, Wann-Hansson C. Prevalence and incidence of domestic violence during pregnancy and associated risk factors: a longitudinal cohort study in the south of Sweden. BMC Pregnancy Childbirth. 2016;16:1–10. 27530993 10.1186/s12884-016-1017-6 PMC4988038

[37] Cox JL, Holden JM, Sagovsky R. Detection of postnatal depression. Development of the 10-item Edinburgh postnatal depression scale. Br J Psychiatry. 1987;150:782–6. 3651732 10.1192/bjp.150.6.782

[38] Eberhard-Gran M, Eskild A, Tambs K, Schei B, Opjordsmoen S. The Edinburgh postnatal depression scale: validation in a Norwegian community sample. Nord J Psychiatry. 2001;55(2):113–7. 11802908 10.1080/08039480151108525

[39] Gibson J, McKenzie-McHarg K, Shakespeare J, Price J, Gray R. A systematic review of studies validating the Edinburgh postnatal depression scale in antepartum and postpartum women. Acta Psychiatr Scand. 2009;119(5):350–64. 19298573 10.1111/j.1600-0447.2009.01363.x

[40] Murray D, Cox JL. Screening for depression during pregnancy with the Edinburgh depression scale (EDDS). J Reprod Infant Psychol. 1990;8(2):99–107.

[41] Lydsdottir LB, Howard LM, Olafsdottir H, Thome M, Tyrfingsson P, Sigurdsson JF. The psychometric properties of the Icelandic version of the Edinburgh postnatal depression scale (EPDS) when used prenatal. Midwifery. 2019;69:45–51. 30396159 10.1016/j.midw.2018.10.009

[42] Spitzer RL, Kroenke K, Williams JB, Löwe B. A brief measure for assessing generalized anxiety disorder: the GAD-7. Arch Intern Med. 2006;166(10):1092–7. 16717171 10.1001/archinte.166.10.1092

[43] Hardardottir D, Vesteinsdottir V, Asgeirsdottir RL, Kristjansdottir H, Thorsdottir F. Próffræðilegir Eiginleikar Íslenskrar Þýðingar GAD-7 í klínísku Úrtaki [in icelandic]. Sálfræðiritið – Tímarit Sálfræðingafélags Íslands. 2022;27:25–39.

[44] Field AP. Discovering statistics using IBM SPSS statistics. London: Sage; 2018.

[45] Sigurvinsdóttir R, Ásgeirsdóttir BB, Arnalds S. Breaking the silence. In: Social media disclosures of sexual violence in Iceland. edn.: Taylor and Francis/Balkema; 2019. p. 224–40.

[46] Ryding EL, Lukasse M, Kristjansdottir H, Steingrimsdottir T, Schei B, Bidens Study G. Pregnant women’s preference for cesarean section and subsequent mode of birth - a six-country cohort study. J Psychosom Obstet Gynecol. 2016;37(3):75–83. 10.1080/0167482X.2016.1181055 27269591

[47] Courtois CA, Courtois Riley C. Pregnancy and childbirth as triggers for abuse memories: implications for care. Birth. 1992;19(4):222–3. 1472273 10.1111/j.1523-536x.1992.tb00408.x

[48] Rachana C, Suraiya K, Hisham AS, Abdulaziz AM, Hai A. Prevalence and complications of physical violence during pregnancy. Eur J Obstet Gynecol Reprod Biol. 2002;103(1):26–9. 12039459 10.1016/s0301-2115(02)00022-2

[49] Alvarez-Segura M, Garcia-Esteve L, Torres A, Plaza A, Imaz ML, Hermida-Barros L, San L, Burtchen N. Are women with a history of abuse more vulnerable to perinatal depressive symptoms? A systematic review. Arch Womens Ment Health. 2014;17(5):343–57. 25005865 10.1007/s00737-014-0440-9

[50] Finnbogadóttir H, Mellgren C. The degree of suffering among pregnant women with a history of violence, help-seeking, and police reporting. Sex Reprod Healthc. 2017;13:23–8. 28844354 10.1016/j.srhc.2017.05.003

[51] Henriksen L, Vangen S, Schei B, Lukasse M. Sexual violence and antenatal hospitalization. Birth. 2013;40(4):281–8. 24344709 10.1111/birt.12063

[52] Hagstofa Íslands. Bakgrunnur Fjöldi innflytjenda. https://www.hagstofa.is/talnaefni/ibuar/mannfjoldi/bakgrunnur

[53] World Medical Association. Declaration of Helsinki: ethical principles for medical research involving human subjects. JAMA. 2013;310(20):2191–4. 24141714 10.1001/jama.2013.281053

[54] World Health Organization. Global Programme on Evidence for Health P: Putting women first: ethical and safety recommendations for research on domestic violence against women. In. Geneva: World Health Organization; 2001.

